# Phytochemical Analysis of *Centaurea calcitrapa* L. Aerial Flowering Parts Serial Solvent Extracts and Its Antibacterial and Antioxidant Activities

**DOI:** 10.3390/life14070900

**Published:** 2024-07-19

**Authors:** Alsayed E. Mekky, Ebrahim Saied, Eslam S. Abdelmouty, Muhammad I. Haggag, Mohamed Khedr, Ashjan F. Khalel, Mahmoud M. Al-Habibi, Shimaa A. Metwally, Ahmad El Askary, Abeer Mahmoud Mohammad, Wafa A. Alshehri, Ahmed I. Sharahili, Nehal M. Khairy, Ahmed E. M. Abdelaziz, Nashaat N. Mahmoud

**Affiliations:** 1 Botany and Microbiology Department, Faculty of Science, Al-Azhar University, Cairo 11884, Egypt; hema_almassry2000@azhar.edu.eg (E.S.); drislam20013@azhar.edu.eg (E.S.A.); muhammadaly@azhar.edu.eg (M.I.H.); mohamedkhedr.221@azhar.edu.eg (M.K.); nashaat_mahmoud@azhar.edu.eg (N.N.M.); 2 Biology Department, Al-Darb University College, Jazan University, Jazan 45142, Saudi Arabia; akhalel@jazanu.edu.sa (A.F.K.); ayousuf@jazanu.edu.sa (A.M.M.); 3 Microbiology and Immunology Department, Faculty of Pharmacy (Boys), Al-Azhar University, Cairo 11884, Egypt; mahmoudalhabibi@azhar.edu.eg; 4 Microbiology and Immunology Department, Faculty of Pharmacy (Girls), Al-Azhar University, Cairo 11884, Egypt; dr.shimaaabdelrahman@azhar.edu.eg; 5 Department of Clinical Laboratory Sciences, College of Applied Medical Sciences, Taif University, P.O. Box 11099, Taif 21944, Saudi Arabia; a.elaskary@tu.edu.sa; 6 Department of Biological Sciences, College of Science, University of Jeddah, Jeddah 23890, Saudi Arabia; waalshehri@uj.edu.sa; 7 Department of Clinical Laboratories, Medical Biochemistry Unit, Najran General Hospital, Najran 66277, Saudi Arabia; aisharahili@moh.gov.sa; 8 Ministry of Health, Riyadh 12613, Saudi Arabia; 9 Department of Microbiology and Immunology, Egypt Drug Authority (EDA), (Formerly NODCAR), Giza 12654, Egypt; nehal.khairy@su.edu.eg; 10 Department of Microbiology and Immunology, Faculty of Pharmacy, Sinai University—East Kantara Branch, Ismailia 41636, Egypt; 11 Botany and Microbiology Department, Faculty of Science, Port-Said University, 23 December Street, P.O. Box 42522, Port-Said 42522, Egypt; ahmed.abdelaziz@sci.psu.edu.eg

**Keywords:** antibacterial, antioxidant, *Centaurea calcitrapa* L., phytochemical analysis, scavenging activity

## Abstract

To evaluate the phytochemical composition, antibacterial, and antioxidant activity of successive extracts of *Centaurea calcitrapa* L. (*C. calcitrapa*) aerial flowering parts, they were assessed in vitro. Using a spectrophotometer, the sample absorbance at 517 nm was used to quantify the scavenging activity. The negative control was DPPH. In the current study, the diffusion using agar wells technique was adapted to measure antimicrobial activity. Phytochemical analysis was performed using the recommended standard procedures. The methanol extract of *C. calcitrapa* exhibited high levels of total phenolic acids expressed as gallic acid (GA), measured as (97.25 ± 0.73 mg GAE/g) content compared to the chloroform, acetyl acetate, and aqueous extracts (27.42 ± 0.29, 64.25 ± 0.96, and 17.25 ± 0.73 mg GAE/g), respectively. Additionally, the methanol extract had a higher total tannin (27.52 ± 0.53 mg TAE/g) content compared to the chloroform, ethyl acetate, and aqueous extracts (12.02 ± 0.55, 26.01 ± 0.81, and 7.35 ± 0.56 mg TAE/g), respectively, while the aqueous extract contains a lower percentage of flavonoids (141.10 ± 1.31 mg RTE/g) compared to the higher content achieved by the methanol extract (425.93 ± 1.27 mg RTE/g). The hydroxyl groups of the flavonoid and the phenolic compounds found in *C. calcitrapa* are essentially scavenging free radicals. Radical scavenging activity was highest in the methanol extract (IC50 = 2.82 μg/mL), aqueous extract (IC50 = 8.03 μg/mL), ethyl acetate extract (IC50 = 4.79 μg/mL), and chloroform extract (IC50 = 6.33 μg/mL), as compared to the standard scavenging activity (IC50 = 2.52 μg/mL). The antibacterial properties of *C. calcitrapa* against Gram-negative bacterial strains *Klebsiella pneumoniae*, *Escherichia coli*, *Enterobacter aerogenes*, and *Acinetobacter baumanii*, in addition to Gram-positive strains *Staphylococcus haemolyticus*, *Enterococcus faecalis*, and *Staphylococcus aureus*, revealed inhibition zone diameter. The findings of this investigation establish that the aerial flowering parts of *C. calcitrapa* have substantial antibacterial action against human infections, and the plant can serve as a significant antioxidant that can be employed to prevent and treat severe degenerative diseases brought on by oxidative stress. qPCR showed that *C. calcitrapa* extracts elevate both SOD1 and SOD2 (cellular oxidation markers) with remarkable folds (1.8-fold for SOD1 and SOD2) with ethyl acetate plant extract against ascorbic acid as a control. This result reflects that *C. calcitrapa* extracts have remarkable antioxidant activity.

## 1. Introduction

According to estimates from the World Health Organization, 70% of people worldwide still rely on medicinal plants, which are sources of a variety of active ingredients. Less than 10% of the 25,000 plant species in existence have had their therapeutic properties researched thus far [1]. Phenols or their derivatives with an oxygen substitution make up the majority of the aromatic chemicals that plants may generate [2]. Only 12,000 of the entire quantity of metabolites, or less than 10%, have been identified, with secondary metabolites accounting for the majority [3]. Only a limited portion of them have undergone phytochemical investigation, and even fewer have undergone biological or pharmacological testing. Since plants may contain hundreds or even thousands of metabolites, there is currently a lot of interest in medicinal plant research as a possible source of novel lead compounds for entry into therapeutic screening programs [4]. The objective of the emerging scientific field of ethnobotany (also known as ethnopharmacology), which aims to make use of the amazing body of information gathered by original peoples on the animal and plant products they have employed to sustain healthiness [5], is to utilize this knowledge. Undoubtedly, one of the worst causes of mortality is bacterial infection [6]. Antibiotic treatment is essential for recovery. However, the incidence of bacterial infections that are resistant to several drugs is rising over time as a result of overuse and incorrect use of antibiotics [7]. Traditional medical approaches and common antibiotics have little effect. Antibiotic resistance is an important issue in terms of community health [8]. Due to the limited number of, and occasionally nonexistent, effective medicines available for illnesses brought on by these bacteria, the rise of multidrug-resistant microorganisms has limited the use of antibiotics in the medicine [9]. To direct the development, discovery, and research for novel medications, the global priority list of bacteria resistant to antibiotics was created by the World Health Organization (WHO) in 2017 [10]. This list includes *A. baumannii*, *K. pneumoniae*, *E. faecium*, *Helicobacter pylori*, *Pseudomonas aeruginosa*, *S. aureus*, *Shigella* spp., *Salmonella* spp., and so forth [11,12]. It implies that these bacteria urgently require new antibiotics. However, the creation of new antibiotics was hampered by high costs (several hundred million dollars), unpredictability (typically ten years), and commercial failure (just 17 novel antibiotics were authorized between 2010 and 2021) [13]. Researchers must devise new strategies as the strain increases. Researchers are interested in natural compounds made from plants because they may be able to manage germs that are resistant to antibiotics [14,15]. Human diseases have historically been treated using plants as a source of medicine, and between 70 and 95 percent of people in some underdeveloped countries still use plants as their major source of medication [16]. Long-term evolution promotes the competition for survival between dangerous bacteria and plants. Plants create a variety of secondary metabolites during this process to protect themselves from the invasion of harmful microorganisms [17]. Additionally, microbial biofilms play a crucial role in several illnesses, and biofilm-related traits may cause microbial populations to exhibit high levels of antibiotic resistance [18]. Numerous targeting receptors and bioactivities result from the immense chemical and structural variety of plants, which may make up for the dearth of novel antibacterials [19]. Many different antioxidants or free radical-scavenging substances, including phenols, terpenoids, vitamins, and flavonoids, are present in plants with high antioxidant capacity [20]. Plant-derived polyphenolic components may have better effects in vivo than vitamins E or C because they are more potent antioxidants in vitro [21]. *C. calcitrapa* is a 60 cm high biennial herbaceous perennial plant that is also known as purple star thistle. It can be found in Western Asia, Northwestern India, North Africa, and Western and South-Central Europe. It thrives on rocky terrain, rich soil, sunny slopes, and warm climates. It also spreads between railroad tracks and along roadside ditches [22]. Numerous Centaurea species have been employed as traditional medicines for hundreds of years to diagnose and treat many different kinds of diseases, including cancer, anorexia, diabetes, inflammation, fever, diarrhea, digestive disorders, rheumatoid arthritis, microbial infections, and others [23]. With more than 600 species found all over the world, mainly in Western Asia and the Mediterranean, the fourth major genus in the Asteraceae family is *Centaurea.* In Egypt’s Red Sea, Nile, and Mediterranean coastal regions, there are about 17 different species of *Centaurea.* Previous research on several plant extracts has revealed some bioactivity potential [24]. Aqueous and methanol (MeOH) extracts have been shown to have significant cytotoxic action on the HeLa (cervical cancer in humans) and Vero (renal epithelial cells from an African green monkey) cell lines, as well as substantial antioxidant properties [25]. The methanol extract has demonstrated potent antibacterial activity against a variety of pathogens, including members of the *Bacillus*, *Staphylococcus*, *Pseudomonas*, *Salmonella*, *Streptococcus*, *Acinetobacter*, *Enterobacter*, *Enterococcus*, and *E. coli* genera [26]. Sterols and sesquiterpene lactones, as well as a closely related group of triterpenoids, lignans, flavonoids, and bisabolene, were found in *C. calcitrapa* extracts, according to several phytochemical studies [25]. Therefore, it is crucial to classify the various types of therapeutic plant compounds according to their antioxidant and antibacterial properties [27]. Products made from medicinal plants offer several advantages over manufactured chemical substances, including activity, fewer side effects, a reduced price, and availability [28]. Therefore, the study aimed to identify appropriate solvents for extraction to determine the screening for phytochemicals, quantitative of total phenol, flavonoid, tannins, antibacterial, and antioxidant action of *C. calcitrapa* aerial flowering parts. The results we obtained provide an initial basis for further research on the isolation, characterization, and potential applications of active compounds in drug development.

## 2. Material and Methods

### 2.1. Solvents and Chemicals

High-purity chemicals, including DPPH, dimethylsulfoxide (DMSO), ferric chloride, NaOH, HCl, H_2_SO_4_, and AlCl_3_, were purchased from Sigma-Aldrich; in Cairo, Egypt. Analytical-grade solvents were acquired from Sigma-Aldrich. Mueller–Hinton agar and PDA agar were purchased from Hi Media in Cairo, Egypt.

### 2.2. Sample Preparation

At the noted location (31324.11276° N 281310.69781° E), the aerial flowering parts of *Centauraea calcitrapa* L. were collected from 40M, El Dabaa, Matrouh Governorate, Egypt. *C. calcitrapa* fresh aerial flowering parts were distilled water-cleansed, then dried in the shade at lab temperature until they had a constant weight. After being pounded into a fine powder and sieved, the dried plants were kept at room temperature in dry glass jars for future use. 

### 2.3. Preparation of Plant Extracts

The powdered and air-dried aerial flowering parts of *C. calcitrapa* (100 g) were subjected to successive extraction using various organic solvents based on their polarity, starting with chloroform, ethyl acetate, methanol, and aqueous using a Soxhlet apparatus at a temperature lower than the boiling point of the solvents. To create the crude-dried extracts, the extracts were freeze-dried after being condensed under decreased pressure in a rotary evaporator. For antibacterial investigations, the extracts were dissolved in DMSO at 0.1%. To determine if phytochemicals are present or absent in plant crude extract, phytochemical screening is often conducted. In most cases, results are either stated as present (positive [+]) or missing (negative [−]). They were repeatedly extracted using the cold extraction procedure with water, methanol, ethyl acetate, and chloroform. Rotating evaporators were used to concentrate the extracts and for phytochemical analysis.

### 2.4. Phytochemical Screening Tests

Phytochemical analysis of the plant extracts was carried out qualitatively using the methods described by Harborne [29] to look for the presence of phenolic acids, alkaloids, quinines, flavonoids, steroids, saponins, tannins, anthraquinone, cardiac glycosides, phlobatannins, carbohydrates, and fixed oils.

### 2.5. Total Phenolic Acid Content

Total phenolic acid content was estimated by the Folin–Ciocalteu process explained by Makkar [30]. A 50 µL amount of phenolic extract should be divided among several test tubes. Fill each test tube’s contents to 1 mL using distilled water. To each tube, including the blank, add 0.5 mL of Folin–Ciocalteu reagent (1 N). Then, allow it to sit for 5 min at room temperature. Finally, add 2.5 mL of all the tubes, including the blank, with Na_2_CO_3_ (5%). After that, incubate for 40 min at room temperature in a dark place. A spectrophotometer was used to detect the 725 nm absorbance of the calibration curve for gallic acid, which was used as the standard that had been dissolved in methanol.

### 2.6. Total Flavonoid Content

Total flavonoid was determined by the aluminum chloride procedure designated by Zhishen et al. [31]. Take 500 µL of the extract into a series of test tubes. Fill every experiment tube’s content to 1000 µL with distilled water. Then, add 150 µL of NaNO_2_ (5%) to all tubes and incubate for 5 min at room temperature. Add 150 µL of AlCl_3_ (10%) to all the test tubes and incubate for 6 min. at room temperature. Add 2 mL of NaOH (4%) to all the tubes. Fill tubes to a volume of 5 mL with distilled H_2_O. The test tubes vortex and allow standing at room temperature for 15 min. As the standard was dissolved in methanol and examined by a spectrophotometer at 510 nm, the rutin calibration curve was employed.

### 2.7. Total Tannin Content

The content of tannins was evaluated using the Folin–Denis spectrophotometer process [30]. The tannins were precipitated by adding 0.5 mL of plant sample and 0.5 mL of distilled water to 0.1 g of polyvinyl polypyrrolidone in a cold environment. The tubes were then incubated at 4 °C for 4 h before being centrifuged for 10 min. Only the non-tannin phenolics are present in the supernatant. Take 0.5 mL of the Folin–Ciocalteu reagent (1 N) and 100 μL of the sample’s non-tannin phenolic extract. With distilled water, fill each test tube, including the blank, to a volume of 1 mL. After thoroughly combining all the tubes, let them rest at room temperature for 5 min. Then, add 2.5 mL of Na_2_CO_3_ (5%), including the blank, to all the test tubes. Mix the tubes again and incubate for 40 min. at room temperature in the dark. The calibration curve of tannic acid used as the standard dissolved in methanol was measured by a spectrophotometer at 725 nm.

### 2.8. Biological Studies

#### 2.8.1. Testing Organisms

All the human pathogen microbial strains used in the antimicrobial assay have been obtained from Al-Azhar University’s Regional Centre for Mycology and Biotechnology in Nasr City, Cairo, Egypt. Gram-negative bacteria, including *K. pneumoniae*, *E. coli*, *E. aerogenes*, and *A. baumanii*, are examples of these pathogenic organisms; Gram-positive bacteria, including *S. haemolyticus*, *E. faecalis*, and *S. aureus*, were all identified through the VITEK2 system. The activity was calculated as a function of the inhibition zone in millimeters (mm).

#### 2.8.2. Method for Diffusing Agar Wells

As pointed out by Saied et al. [32], as test specimens, many human pathogenic microorganisms were used. In nutrient broth, pure cultures of the test specimens were subcultured for bacteria. On Mueller–Hinton agar-coated, sterile petri plates, the strains were evenly distributed. A sterile cork borer was used to make a circular borehole in plates that were 6 mm in diameter. To test the antimicrobial activity, 100 μL of various *C. calcitrapa* extracts were transferred to the well. The plates were subsequently incubated at 37 °C overnight, and the zones of inhibition were determined. The positive control was a typical antibiotic for bacteria (Amoxicillin/Clavulanic acid (AMC)) at a concentration of 2000 μg/mL, while the negative control was sterilized distilled water. Microbial growth was detected visually. Each test has been performed three times.

#### 2.8.3. Determination of Minimum Inhibitory Concentrations (MICs)

The MBCs and MICs of different *C. calcitrapa* extracts were prepared using the method defined in the guidelines of the Clinical Lab Standards Institute (US) Method. MHA plates have been employed for the MBC test; the MIC test was performed on 96-well round-bottom microtiter plates using conventional broth microdilution techniques. The concentration of the microbial inoculum was changed to 1.5 106 CFU/mL. To conduct the MIC test, 100 μL of various *C. calcitrapa* extract solution stocks (800 μg/mL) were added, diluted twice, and inoculated into 100 mL of MHB, starting from columns 4 to 12. The concentration of the tested components was highest in column 4 and lowest in microtiter plate column 12. As a positive control, column 1 included both media and bacterial inoculums, whereas column 2 contained just medium. Each well of the microtiter plate received 30 μL of the resazurin solution and was incubated for 24 h at 37 °C. No color variations were seen. Pink or colorless showed bacterial development, whereas blue or purple indicated the absence of bacterial growth. The lowest concentration at which there was no color change was found to be the MICs. Each well’s test dilutions that did not change color were subcultured and incubated for 24 h on agar plates, and MBCs were determined. As the lowest concentration has stopped bacterial growth, the MBC values were calculated. The outcomes are displayed in µg/mL [33].

#### 2.8.4. Resazurin Solution

According to Khalifa et al. [34], at 0.02% (wt/vol), a resazurin solution was produced. Next, 0.002 g of resazurin salt powder was immersed in 10 mL of distilled water and then subjected to a vortex. The mixture was filtered by a Millipore membrane filter (0.2 m). The resazurin solution can be stored at 4 °C for two weeks.

#### 2.8.5. Estimation of Minimum Lethal Concentrations (MLCs)

The MLC of different *C. calcitrapa* extracts against tested pathogens was evaluated using the microbroth dilution test as designated by Mekky et al. [35], with some changes. Every culture was grown in media employing plant extract; the group in line number one without plant extract was taken as a negative control, and the group in line number two without bacteria was taken as a positive control. For the determination of the MLCs, a treatment of twofold dilution at various concentrations (200–1.56 mg/mL) was chosen. To determine the MLC, the overnight-grown cultures from each treatment concentration were subsequently streaked on agar plates.

#### 2.8.6. Antioxidant Activity

##### Estimation of Antioxidant Activity Using the DPPH Radical Scavenging Procedure

Different extracts from the aerial flowering parts of *C. calcitrapa* were evaluated using the DPPH (2,2-diphenyl-1-picryl-hydrazyl-hydrate) procedure for their ability to scavenge free radicals. To put it briefly, a 0.1 mM DPPH solution in ethanol was made. Next, 3 mL of various extracts in ethanol were mixed with 1 mL of this solution at several concentrations (0, 1.95, 3.9, 7.8125, 15.625, 31.25, 62.5, 125, 250, 500, and 1000 μg/mL). In this case, only extracts that are soluble in ethanol are used, and different quantities of those extracts were created using the dilution process. After vigorous shaking, the combination took 30 min to recover at room temperature. Then, using a UV–visible Milton Roy spectrophotometer, absorbance was determined at 517 nm. Three replicates of the experiment were run, using ascorbic acid as the reference standard compound [36]. The sample’s IC50 value, or using a log dosage inhibition curve, the concentration of the sample essential to inhibit 50% of the DPPH free radical, was established. A reaction mixture with lower absorbance was a sign of increased production of free radicals. The percentage of the evaluation of the DPPH scavenging effect using the following equation is:

Effect of DPPH scavenging (%) or percent inhibition (%) = A_0_ − A_1_/A_0_ × 100

where: A1 was the absorbance when the test or reference sample was present, and A0 was the control reaction’s absorbance.

##### Ferric Reducing/Antioxidant Power (FRAP) Assay

Benzie and Strain’s [37] methodology, with a few experimental adjustments, guided the use of the FRAP test to evaluate the antioxidant capacity of plant extracts. The test was predicated on a sample’s reducing power. By converting the ferric ion (Fe^3+^) to the ferrous ion (Fe^2+^), a possible antioxidant will create a blue color complex (Fe^2+^/TPTZ), which would boost absorption at 593 nm. A solution of 10 mM of TPTZ in 40 mM of HCl, 20 mM of FeCl3, and 300 mM of acetate buffer (pH 3.6) were combined to create the FRAP reagent at a ratio of 10:1:1 (*v*/*v*/*v*). An amount of 600 μL of newly made FRAP reagent and 1.0 mL of distilled water with varying concentrations of plant extracts (50 μg–300 μg) make up the reaction mixture. After carefully mixing the reaction mixture, it was left in the dark for 30 min. At 593 nm, absorbance was measured. The standard calibration curve was created with FeSO_4_.7H_2_O concentrations that were known. The standard antioxidant, ascorbic acid, was utilized, and the antioxidant activity was represented in terms of equivalents of ascorbic acid. Every determination was carried out three times.

Relative % of reducing power = (A − A_min_)/(A_max_ − A_min_) × 100

where A—Abs of Sample, A_min_—Abs of control, A_max_—Highest Abs of Standard.

##### Hydrogen Peroxide Scavenging Activity

With minor experimental adjustments, the Ruch et al. [38] investigation was used to assess the plant extracts’ capacity to scavenge hydrogen peroxide. A 40 milligram hydrogen peroxide solution (pH 7.4) was made in 0.1 M of phosphate buffer. A 0.6 mL quantity of a 40 mM hydrogen peroxide solution was mixed with 1.0 mL of distilled water containing varying amounts of plant extracts (50 μg–300 μg). The absorbance of hydrogen peroxide at 230 nm was measured after 10 min of incubation and compared to a blank solution that included phosphate buffer but no hydrogen peroxide. The standard antioxidant utilized in this study was ascorbic acid, and the hydrogen peroxide scavenging activity was quantified in terms of ascorbic acid equivalents. Every determination was carried out in three copies. The proportion of standard compounds and extracts that scavenged hydrogen peroxide was computed:

% Scavenged [H_2_ O_2_] = [(Ac − As)/Ac] × 100

where Blank—Containing only phosphate buffer without hydrogen peroxide, Ac—absorbance of the control without samples/standard, As—absorbance presence of the sample/standards.

##### Reducing Power Assay

With certain experimental changes, Oyaizu et al.’s technique [39] as described in [40] was used to investigate the reducing capabilities of plant extracts. One milliliter of 0.2 M sodium phosphate buffer (pH 6.6) with varying concentrations (50 μg–300 μg) of plant extracts and one milliliter of 1% potassium ferricyanide (*w*/*v*) make up the reaction mixture. For twenty minutes, the mixture was incubated at 50 °C. Following room temperature cooling, 1.0 mL of 10% *w*/*v* trichloroacetic acid was added, and the mixture was centrifuged for 10 min at 3000× *g*. The absorbance was measured at 700 nm after the top layer (1.0 mL) was combined with 1.0 mL of distilled water and 0.4 mL of 0.1% ferric chloride. The standard utilized was ascorbic acid. Phosphate buffer was created in blank form, and neither a standard nor a test substance were added. Greater absorbance is a sign of the sample’s stronger reducing power. The assays were run three times.

Relative % of reducing power = (A − A_min_)/(A_max_ − A_min_) × 100

where A—Abs of Sample, A_min—_Abs of control, A_max_—Highest Abs of Standard.

#### 2.8.7. 16S Identification of MDR Bacterial Isolates

Whole bacterial genomic DNA was extracted from all the tested bacterial isolates. DNA purity and concentration were adjusted through nanodrop, and then the 16S rDNA gene was amplified through two bacterial universal primers: the RW primer (CCAGCCGCAGGTTCCCCT) and the 16Sb FW primer (CCGTGGCGGCAGGCTTAACA) [41]. PCR conditions were set as follows: total 30 cycles; 95 °C/35 s for the denaturation step, 56 °C/40 s for the annealing step, 74 °C/42 s for the extension step, and 72 °C/60 s for the final extension. The amplified PCR product size was estimated through [1.5%] agarose gel. Then, PCR products were purified from gel and sequenced using a Perkin Elmer 377 DNA sequencer in conjunction with a Dye Deoxy Terminator Cycle Sequencing Kit (PerkinElmer, Foster City, CA, USA) [42].

#### 2.8.8. Ribonucleic Acid Isolation

Total ribonucleic acids (RNAs) were extracted from the hepatic cell line of Homo sapiens, and reverse transcription was performed for the synthesis of cDNA for the sense strand through the Advantage RT-PCR Kit (Clontech, Alo Alto, CA, USA).

#### 2.8.9. Conventional Reverse Transcription Polymerase

Chain Reaction Amplification of 50 µL reactions of polymerase chain reactions PCR was conducted with the primers in Table 1 for the SOD1 and SOD2 genes along with the glyceraldehyde-3-phosphate dehydrogenase gene as a housekeeping gene. Primers were used in this study and were created using the NCBI site’s Primer BLAST tool.

The PCR reaction mixture consists of 100 ng of cDNA, 1 mM of each FW and RW primer, 195 mM of dNTPs, 1.7 mM of MgCl_2_, one unit of Taq DNA polymerase (Takara, Tokyo, Japan), and then injection water was added to adjust the volume to 50 µL. The following describes the PCR program of 35 cycles:

Denaturation at 93 °C/2 min at the first cycle; other cycles were at 93 °C/35 s denaturation, annealing at 59 °C/30 s, and extension step at 72 °C/1 min. The size of products was determined using a standard-length DNA ladder (GeneRulerTM 100 bp DNA Ladder, MBI Fermentans, Vilnius, Lithuania).

#### 2.8.10. Real-Time PCR Amplification

In regards to the primers listed in Table 1, complementary DNA (cDNA) samples were subjected to semiquantitative PCR. The PCR mixture consisted of 12.5 µL of 2× Quanti tech SYBR^®^ Green RT Mix (Fermentase.com (accessed on 8 February 2024)), 2 µL of 25 pm/L FW and RW primers, 1 µL of 50 ng cDNA, and 9.25 µL of RNase-free water. The initial denaturation step in PCR real-time programs lasts for 15 min at 95 °C for 30 cycles of 15 s; annealing for 35 s at 59 °C and extending for 42 s at 72 °C.

## 3. Statistical Analysis

The data were analyzed with SPSS version 19 and represented as the mean and standard deviation of three replicates. To determine the differences between means, a Tukey post hoc one-way ANOVA was employed. Significant values were defined as those with a *p* < 0.05. The qPCR result was analyzed using the dd∆ct technique and Microsoft Excel 2016. A comparative quantification analysis was carried out.

## 4. Results and Discussion

Table 2 displays the color of the *C. calcitrapa* extracts. Different extraction methods using chloroform, ethyl acetate, methanol, and aqueous extract were used to retrieve the aerial flowering parts. The aerial flowering parts exhibit various extract colors, including yellow-brown, green, dark green, and dark brown, when dissolved in chloroform, ethyl acetate, methanol, and aqueous, respectively. The data observe the yield residual after the extraction. For the creation of novel chemotherapeutic drugs, plants constitute a significant source of functional components. Since the genus *Centaurea* has known medicinal promise, it has attracted a lot of attention in the search for and development of innovative medication formulations [43].

The phytochemical analysis of the aerial flowering parts of *C. calcitrapa* revealed the presence of flavonoids, saponins, sterols, tannin, glycosides, phenolic acids, quinone, alkaloids, and carbohydrates (Table 3), while it yielded negative results for fixed oils and fats, phlobatannins, resins, cardiac glycosides, and anthraquinone. In all four extracts, according to phytochemical screening, there are carbohydrates, flavonoids, tannins, and phenolics present, whereas methanol, aqueous, and ethyl acetate extracts had favorable results for glycosides. Methanol and water extracts both contain significant amounts of saponins and quinones. Ethyl acetate and chloroform extract do not include them. Additionally, chloroform and ethyl acetate extracts contain significant amounts of sterols and terpenes. Alkaloids are also only highly concentrated in methanol extract. Among the extracts analyzed, there are differences in the secondary metabolite concentration. These components are present in these extracts, indicating that they may have some therapeutic use. This is most likely because each of the detected components has a history of being used therapeutically in one way or another. Phytochemicals have been utilized as medicines for countless years because they frequently play a crucial role in a plant’s defense against predators, microorganisms, stress, and interspecies threats. Thus, the first step in determining the kinds of probable active molecules in plants is phytochemical screening [44].

The phytochemical screening was determined to evaluate the total phenolic acids, flavonoids, and tannins content of aerial flowering parts by different solvents (Table 4). The results revealed that the total phenolic content of the methanol extract was higher and expressed as gallic acid (GA), measured as (97.25 ± 0.73 mg GAE/g) content compared to the chloroform, acetyl acetate, and aqueous (27.42 ± 0.29, 64.25 ± 0.96, and 17.25 ± 0.73 mg GAE/g), respectively, while the aqueous extract had a lower flavonoid content (141.10 ± 1.31 mg RTE/g) compared to the higher content achieved by the methanol extract (425.93 ± 1.27 mg RTE/g). Additionally, the methanol extract had a higher total tannin (27.52 ± 0.53 mg TAE/g) content compared to the chloroform, ethyl acetate, and H_2_O (12.02 ± 0.55, 26.01 ± 0.81, and 7.35 ± 0.56 mg TAE/g), respectively. The total phenolics of the *Aloe vera* rind were found to gradually decrease, from (275.90 ± 20.19 mg GAE/100 g DW) found in the wet sample to (223.99 ± 20.83 mg GAE/100 g DW) in the sample that was heated and air-dried, according to Ng et al. [45]. The fresh sample had the lowest amount of flavonoids (208.70 ± 934.26 mg QE/100 g DW), whereas the freeze-dried sample had the maximum amount (97.10 ± 9.89 mg QE/100 g DW). The bioactive chemical groups known as polyphenols have a variety of positive effects on both the health of humans and animals [46]. Fruits, propolis, and vegetables contain polyphenols called tannins and flavonoids. According to reports, flavonoids function as antioxidants and are essential for the avoidance and treatment of several diseases [47], although tannins have been linked to changes in metabolism and intestinal microbiota [48]. According to the early phytochemical study, the methanolic extract included three significant bioactive substances, namely phenols, tannins, and flavonoids. According to multiple researchers, methanol was the most efficient solvent for flower extraction compared to other solvents [49]. Based on earlier studies and phytochemical discoveries, we extracted bioactive chemicals from *P. pterocarpum* flower petals using methanol in the current investigation, and we saw promising outcomes against seven human diseases [50]. These medicinal plants contain phenol and flavonoid components, which demonstrate their anti-inflammatory and antioxidant-fighting potential.

**Figure 1:**
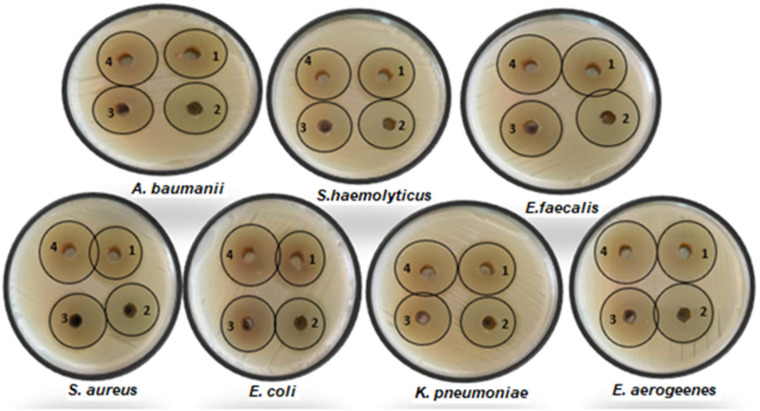
Inhibitory zones of different extractions against pathogenic tested microorganisms, where 1, aqueous Ext.; 2, Chloroform Ext.; 3, Ethyl acetate Ext.; 4, Methanol Ext.

**Figure 2:**
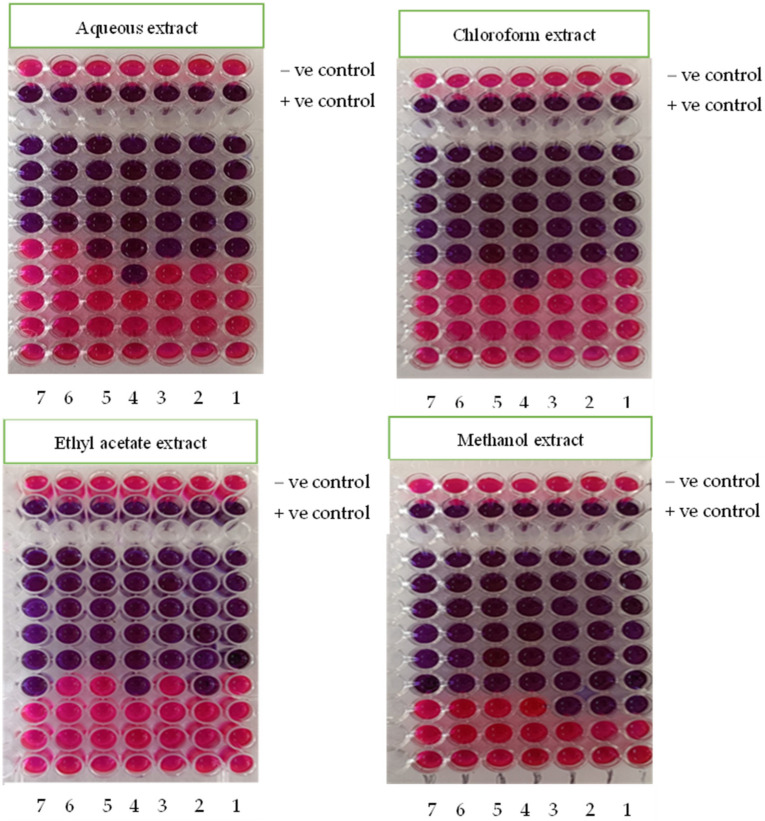
Minimum inhibitory concentrations and minimum lethal concentrations of different extracts of *C. calcitrapa* by Resazurin technique.

The indiscriminate use of commercial antimicrobial medications, which are often utilized in the treatment of illnesses, has caused the development of medication resistance in bacteria that are harmful to humans [51]. This circumstance has compelled scientists to look for novel antibacterial compounds from a variety of sources. The first step in finding novel biomolecules of plant origin and establishing eco-friendly management of infectious illnesses in people is to evaluate plants for their antibacterial properties in vitro. The different extracts of *C. calcitrapa* were tested in vitro for their ability to combat seven human pathogenic microorganisms. The plant is active against both Gram-negative and Gram-positive bacteria, according to the zone of inhibition findings of the current experiment. The antimicrobial activity (Table 5 and Figure 1) of the methanol, chloroform, ethyl acetate, and aqueous extract of aerial flowering parts of *C. calcitrapa* exhibited action against all of the tested bacteria, with a zone of inhibition between 27 and 33 mm at a concentration of 100 μL/mL for each extract. The methanol extract outperformed the other extracts in terms of its antibacterial efficacy against both Gram-positive and Gram-negative bacteria. The inhibition zone against both Gram-positive and Gram-negative bacteria was measured to be between 31 and 33 mm. When applied as a control, the extraction solvents exhibited no activity. Standard antibiotics were also employed in comparison to the extracts, as shown in Table 5. At a dosage of 100 μL/mL, the highest zone of inhibition against the bacterial strains was revealed by a chloroform and ethyl acetate extract to be between 29 and 32 mm. Nevertheless, knowing that the current finding showed that the extracts are efficient against both Gram-positive and Gram-negative bacteria, Gram-positive bacteria are typically thought to be more sensitive than Gram-negative bacteria to various antibacterial compounds due to the modification in the structure of their cell walls. Microorganisms’ development and metabolism are often inhibited by the active ingredients, which also shield them from contamination [52]. Solvent extracts’ greatest efficacy against bacterial strains may be connected to the presence of such phytochemicals. Numerous phenolic substances, such as tannins, are powerful inhibitors of the hydrolytic enzymes utilized by plant pathogens [53]. This plant has antibacterial potential because of its bioactive components, which are recognized to be pesticidal, bactericidal, and fungicidal and are naturally present in most plant materials. Numerous plants emit phenolic substances that are poisonous to microbial infections [54]. Typically, plant-oriented antimicrobial medicines work by lysing the cell walls and membranes of microorganisms, which results in the liberation of cellular content, the inactivation of enzymes, the disruption of protein-binding domains, and eventually the death of the cells [55]. According to earlier findings, *P. pterocarpum* flower extracts have an antibacterial effect (4/3) against four Gram-positive infections and three Gram-negative pathogens [56]. Additionally, the minimal lethal concentrations and minimum inhibitory values of various *C. calcitrapa* extracts against the pathogenic bacteria are illustrated in Table 6 and Figure 2. Following the incubation time, the color change was investigated. A shift from purple to pink, or colorlessness, is a clear sign that the bacteria are actively metabolizing. The lowest concentration at which the color change took place was determined to be the MIC value. With an average of three results, the MICs of the test material and the bacterial strain were calculated [57]. The methanol extract shows MIC values ranging from 6.25 to 12.5 μg/mL, respectively, while the aqueous extract shows that the MIC concentration ranges from 12.5 to 50 μg/mL. The MLCs of pathogenic bacteria against the methanol extract show lethal growth ranging from 12.5 to 25 μg/mL, but the MLCs of pathogenic bacteria against the water and chloroform extracts show lethal growth ranging from 25 to 50 μg/mL.

**Figure 3:**
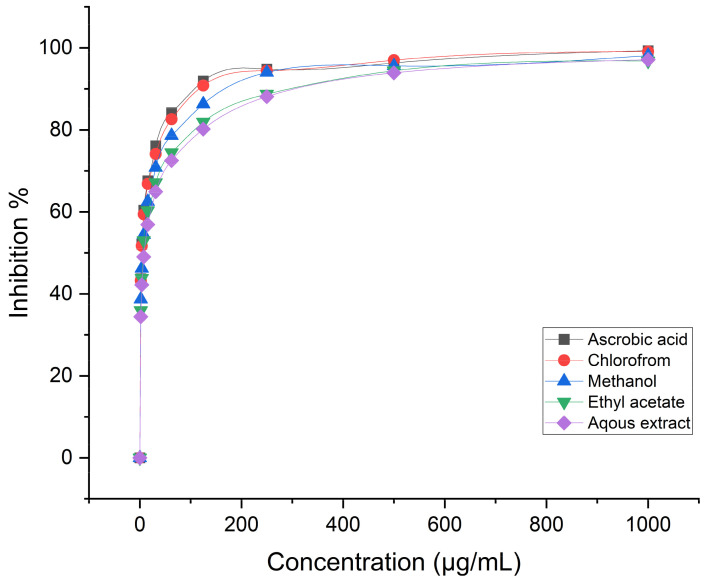
Antioxidant activity of ascorbic acid (AA; positive control), methanol, chloroform, ethyl acetate, and aqueous extract of *C. calcitrapa* at different concentrations.

Evaluation of the medicinal herb *C. calcitrapa* antioxidant capacity in numerous human disorders, including diabetes, multiple sclerosis, Parkinson’s disease, cardiovascular disorders, and respiratory ailments indicates that they are brought on by free radicals [58]. Antioxidant substances are therefore utilized to lessen the impact of free radicals [21]. The aerial floral parts of *C. calcitrapa* extracts showed considerable DPPH scavenging activity in the current study’s free radical scavenger activity tests. The results of all the fractions were found to be significant (*p* < 0.05). In the current study, the antioxidant properties of ascorbic acid (AA), methanol, chloroform, ethyl acetate, and an aqueous extract of *C. calcitrapa* were evaluated at different concentrations from 1000 to 1.95 µg/mL, as shown in Figure 3. In this experiment, DPPH solution was used instead of other free radicals such as superoxide and hydroxyl ions due to the absence of adverse effects like metal ion chelation or enzymatic inhibition [59]. Results showed that, as compared to AA, methanol extract had considerable antioxidant activity. The antioxidant effect of methanol extract at concentrations of 125–1000 µg/mL was above 90% ± 0.24% for all. Moreover, the IC50 of methanol extract was 2.82 µg/mL, while at concentrations of 7.81, 3.9, and 1.95 µg/mL, it was 59.4, 51.7, and 43.2%, respectively. The antioxidant activity of ethyl acetate extract at concentrations of 250–1000 µg/mL was above 90% ± 0.22% for all. The IC50 of methanol extract was 4.79 µg/mL, while at concentrations of 7.8125, 3.9, and 1.95 µg/mL, it was 54.4, 46.2, and 38.7%, respectively. On the other hand, both chloroform and aqueous extracts of the plant exhibited lower antioxidant activity compared to methanol and ethyl acetate, whose IC50s were 6.33 and 8.03 µg/mL, respectively. According to the plants they gathered, Shubharani et al. [60] showed that the Se NPs biosynthesized from the ethanol extract of bee propolis had antioxidant activity ranging from 78.9 to 358.2 g/mL. Hashem et al. [61] observed that the IC50 of Se NPs was 27.8 µg/mL, synthesized by using pomegranate peel extract. Numerous studies link the terpene chemicals found in plant extracts to antioxidant properties. Antioxidant-based formulations are utilized in the prevention and treatment of several disorders, including cancer, diabetes, Alzheimer’s disease, atherosclerosis, and stroke [62]. Due to the presence of free radical scavengers such as flavonoids, and phenolic compounds, polyphenols and natural antioxidants found in herbs and spices are in charge of suppressing or mitigating the negative effects of oxidative stress [21]. Lipid peroxidation, which is caused by these free radicals, harms DNA and cells. Severe oxidative stress induces antioxidant responses such as superoxide dismutase [63], catalase [8], glutathione-S-transferase (GST), and peroxidase (PO) [64]. Elkarim et al. [65] published similar findings; they claimed that the high content of flavonoids and tannins found in the aerial parts of *S. Grantii* was revealed by NMR analyses and that this was primarily due to the isolated compounds’ high antioxidant activity, which amply explains and supports the high radical-scavenging activity of the methanol extract. Researchers evaluated the potential for enzyme inhibition, antioxidant activity, and phytochemicals in a separate study. They found that the ethyl acetate extract profile of *C. saligna* showed substantial antioxidant and inhibitory potential antagonists of butyryl cholinesterase and cholinesterase, as well as the enzymes glucosidase and cholinesterase [66].

**Figure 4:**
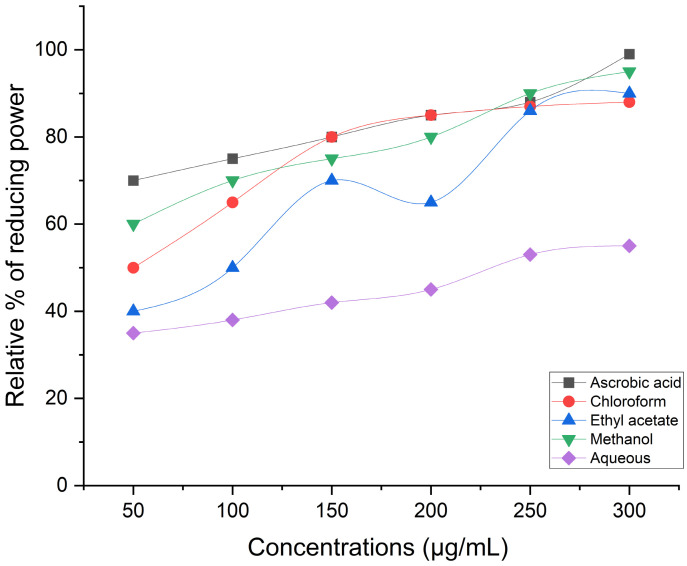
Antioxidant activity-FRAP assay.

**Figure 5:**
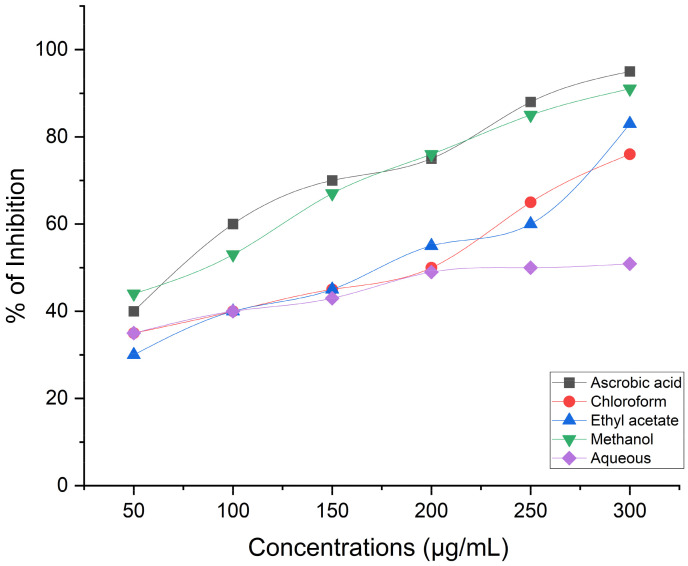
Hydrogen peroxide scavenging activity.

**Figure 6:**
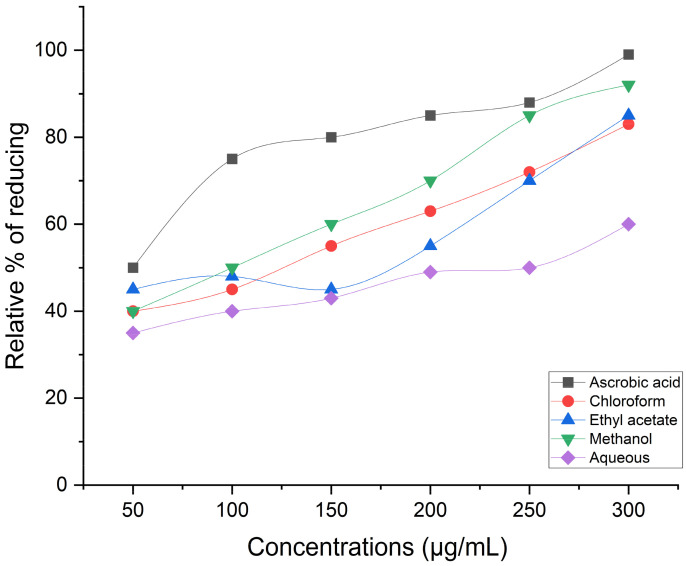
Reducing power assay.

Antioxidants are used as reductants in the FRAP test, which employs a redox-linked colorimetric technique with a complete electron transfer reaction mechanism. This represents the reducing power in relation to ascorbic acid, the standard reference. The greatest relative percent of reducing power was demonstrated at final concentrations by methanol extract (95.1%), followed by ethyl acetate (90%) and chloroform (88.2%). However, the aqueous extract (70.1%) did not provide promising outcomes (Figure 4). When compared to reference standard ascorbic acid, methanol extract (91.25%) has demonstrated a nearly substantial hydrogen peroxide scavenging effect at final concentrations. Good scavenging capacity was demonstrated even for the chloroform extract (76.4%) and the ethyl acetate extract (83.2%). However, distilled water extract (50.9%) shows a modest level of efficient H_2_O_2_ scavenging (Figure 5). The reducing power test, or K_3_ [Fe (CN)_6_] complex, is dependent on antioxidants’ capacity to convert potassium ferricyanide to the ferrous state. The color of the test solution varies to different hues of green, blue, or Perl’s Prussian blue Fe_4_ [Fe(CN)_6_]_3_, which may be determined by spectrophotometrically detecting each sample’s concentration at 700 nm. The reducing power was stated in terms of ascorbic acid, the reference standard. When it comes to potassium ferricyanide, methanol extract (92.30%) has demonstrated excellent reducing capacity (Figure 6).

Keeping in mind the objectives of the current investigations, every antioxidant assay showed significant outcomes. The DPPH test is a frequently used, low-cost, and straightforward approach that is used to reduce a chemical reaction and assess a drug’s antioxidant potential. The DPPH radical is deep violet in solution due to a broad absorption band; when neutralized, it becomes colorless or light yellow. This feature makes it possible to see the response visually. Antioxidants are used as reductants in the FRAP test, which employs a redox-linked colorimetric technique with a complete electron transfer reaction mechanism. Extract from methanol has demonstrated the highest relative percent reduction power. Superoxide dismutase [67] and other monomeric oxidases found in the outer mitochondrial membrane, including amino acid oxidase and xanthine oxidase [68], may dismutate the superoxide anion to produce hydrogen peroxide. Although H_2_O_2_ is not reactive by itself, it may occasionally be very harmful to cells due to its ability to either convert or produce cytotoxic hydroxyl radicals (•OH). It could break down some heat-sensitive proteins, including heat-globin, and oxidize metal ions to produce superoxide-free radicals, such as Fe^2+^ to Fe^3+^, Cu^+^ to Cu^2+^, or what is known as Fenton [69] and Haber–Weiss processes in cells, which grow into extremely potent oxidizing agents. In this instance, methanol extract has demonstrated a nearly substantial ability to scavenge hydrogen peroxide when compared to ascorbic acid, the reference standard. In a redox process, the Fe^3+^/ferricyanide system offers a sensitive “semi quantitative” measurement of diluted polyphenolics [70]. It can also be a useful predictor of an extract’s prospective antioxidant value [71]. Methanol extract has demonstrated potent reducing power when applied to potassium ferricyanide.

**Figure 7:**
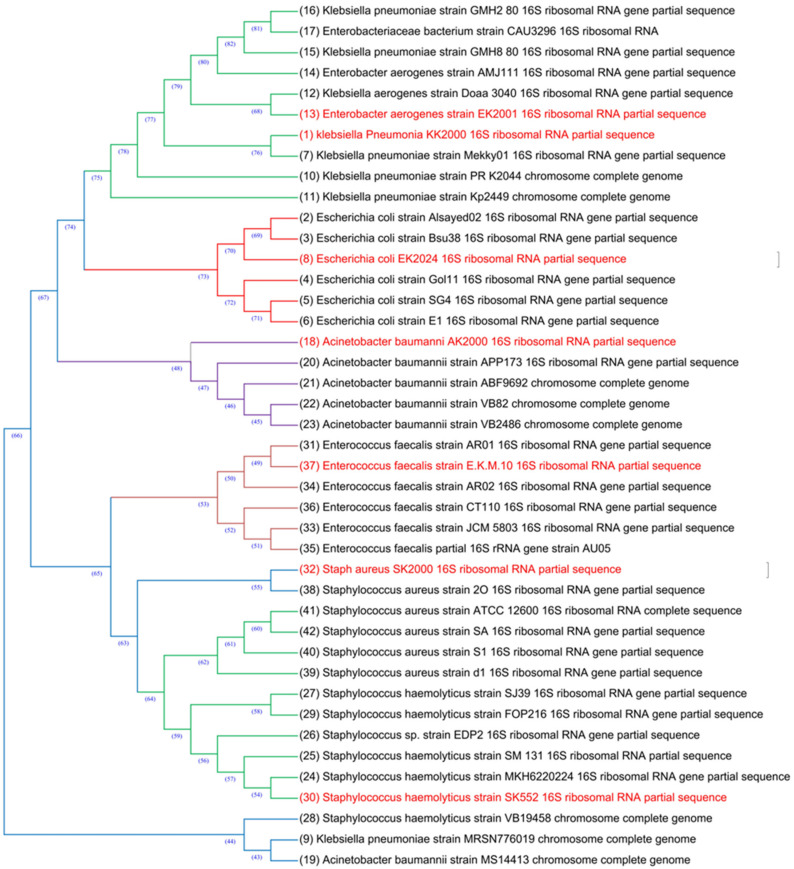
Phylogenetic tree of seven bacterial strains based on 16S sequencing against the most similar sequences for each one through NCBI BLAST.

Test pathogenic bacterial strains were identified though 16S sequencing against the most similar sequences in NCBI GenBank, their description followed by accession numbers as following: *K. pneumoniae* KK2000 (PP789583), *E. coli* EK2024 (PP789584), *E. aerogenes* strain EK2001 (PP789585), *A. baumanni* AK2000 (PP789586), *S. haemolyticus* strain SK552 (PP789587), *E. faecalis* strain E.K.M.10 (PP789588), and *S. aureus* SK2000 (PP789589). Based on 16S sequencing, a BLAST phylogenetic tree for seven bacterial strains is described in Figure 7.

**Figure 8:**
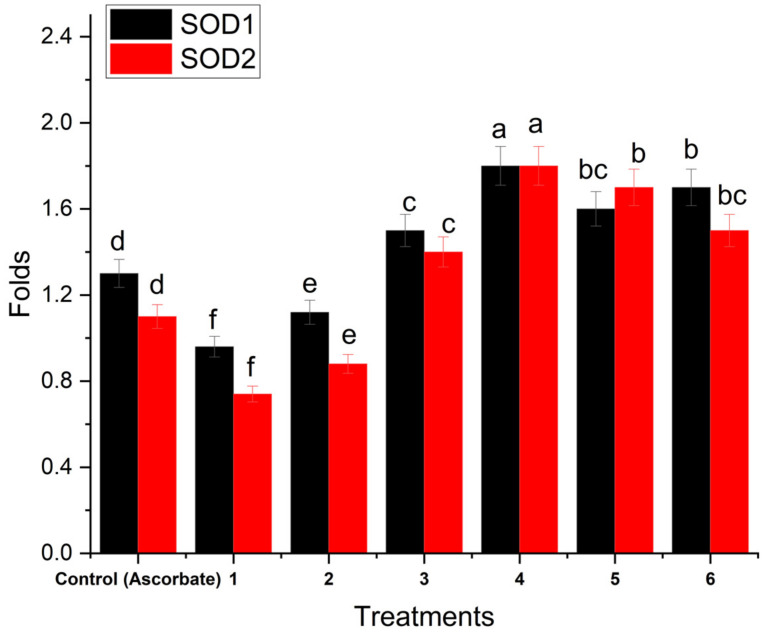
Folds of gene expression through qPCR for SOD1 and SOD2 within hepatocytes cell line treated with plant extracts (3, aqueous ext.; 4, ethyl acetate; 5, chloroform ext.; methanol ext.) and 1, GAPDH-Control; 2, GAPDH-treated. Different letters (a, b, c, d, e, and f) on bars denote that mean values are significantly different (*p* ≤ 0.05) (n = 3).

*C. calcitrapa* (Ethyl acetate extract) exhibits the highest increasing folds for SOD1 and SOD2 with about 1.8-fold for both through qPCR, followed by methanol extract with 1.7- for SOD1 and 1.6-fold for SOD2, followed by aqueous extract with 1.5- for SOD1 and 1.7-fold for SOD2, and chloroform extract with 1.4- and 1.5-fold with SOD1 and SOD2, respectively (Figure 8).

**Figure 9:**
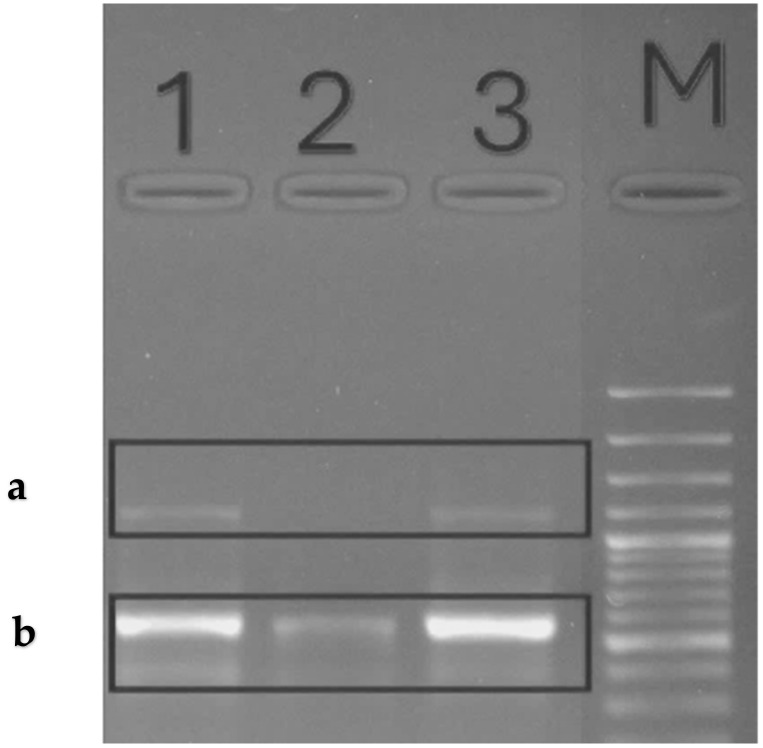
PCR products of SOD1 [270 bps] (**a**) and SOD2 [462 bps] (**b**) against DNA Ladder (M) through 1.5% agarose gel electrophoresis which visualized the estimated size of interest genes. Line 1: methanol extract treated, Line 2: Ascorbic acid treated, while Line 3: ethyl extract treated cells.

Nucleotide sequencing for SOD1, SOD2, and glyceraldehyde-3-phosphate dehydrogenase was carried out through the designed specific primers mentioned in Table 6, PCR products were detected against the GeneRulerTM 100 bp DNA Ladder. The SOD1 PCR product showed a partial sequence with a size estimated at 462 bps, while SOD2 was estimated at 270 bps, as visualized in Figure 9.

## 5. Conclusions

The study demonstrates that different extracts of aerial flowering parts of *C. calcitrapa* exhibit significant antibacterial activities against a spectrum of pathogens, including *E. coli*, *K. pneumoniae*, *E. aerogenes*, *A. baumanii*, *S. aureus*, *S. haemolyticus*, and *E. faecalis*. The methanol extract showed superior antibacterial activity against both Gram-positive and Gram-negative bacteria, with an IC50 value of 2.82 µg/mL. This potency may be attributed to the rich phytochemical profile of *C. calcitrapa*, encompassing flavonoids, alkaloids, phenols, tannins, carbohydrates, and saponins. Furthermore, the extracts exhibited significant DPPH scavenging activity, with the methanol extract showing the highest radical scavenging activity (IC50 = 2.82 µg/mL), followed by the ethyl acetate (IC50 = 4.79 µg/mL), chloroform (IC50 = 6.33 µg/mL), and aqueous extracts (IC50 = 8.03 µg/mL). These findings underscore the potent antioxidant potential of *C. calcitrapa* extracts, which may contribute to their efficacy in combating oxidative stress-related diseases. Additionally, the study demonstrated that *C. calcitrapa* extracts elevate the levels of antioxidant markers SOD1 and SOD2 significantly, particularly with the ethyl acetate extract showing a remarkable fold increase (1.8-fold) compared to ascorbic acid control. This highlights the plant’s potential as a natural source of antioxidants for therapeutic applications. Finally, the aerial flowering parts of *C. calcitrapa* possess substantial antibacterial and antioxidant activities, making them promising candidates for developing natural therapies against bacterial infections and oxidative stress-related disorders.

## Figures and Tables

**Table 1:** Primers were used in this study for two markers, SOD1 and SOD2, beside housekeeping gene (glyceraldehyde-3-phosphate dehydrogenase).

Primer	Sequence (5---------<3)	Tm	GC%	Ref
SOD1-FW	CGTGGCCTAGCGAGTTATGG	60.60	60	This Study
SOD1-RW	ATAGACACATCGGCCACACC	59.82	55	
SOD2-FW	GGCCTACGTGAACAACCTGA	59.97	55	
SOD2-RW	GCCTGTTGTTCCTTGCAGTG	59.97	55	
GPDH-FW	CCGCATCTTCTTTTGCGTCG	60.52	55	
GPDH-RW	TTCCCGTTCTCAGCCTTGAC	59.97	55	

**Table 2:** The yield and color of the various extracts of *C. calcitrapa* aerial flowering parts obtained following extraction with chloroform, ethyl acetate, methanol, and aqueous in succession.

Solvent Used	Sample (Gram)	Boiling Point	Total Hrs of Extraction	Yield (Gram)	Color of Extract
Chloroform	100	60 °C	5	2.96	Yellow-brown
Ethyl acetate	95	68 °C	6	3.02	Green
Methanol	90	80 °C	8	11.24	Dark green
Aqueous	74	95 °C	10	9.09	Dark brown

**Table 3:** The preliminary phytochemical screening of aerial flowering parts of *C. calcitrapa* by successive extracts.

**Constituents**	Tests	Extracts			
		Chloroform	Ethyl Acetate	**Methanol**	Aqueous
Carbohydrates	Molisch’s test	+	+	+	+
Fixed Oils and Fats	Saponification test	−	−	−	−
Phenol	Ferric chloride test	+	+	+	+
Tannins	Ferric chloride test	+	+	+	+
Phlobatannins	HCL test	−	−	−	−
Flavonoids	Lead acetate test	+	+	+	+
	AlCL_3_ test	+	+	+	+
Saponins	Froth test	−	−	+	+
Glycosides	Glycosides test	−	+	+	+
	Conc. H_2_SO_4_ test	−	+	+	+
Alkaloids	Dragendroff’s test	−	−	+	−
	Wagner’s test	−	−	+	−
	Hager’s test	−	−	+	−
Quinone	KOH test	−	−	+	+
Resins	Resins test	−	−	−	−
Sterols and terpenes	Salkowski test	+	+	−	−
Cardiac glycosides	Legal’s test	−	−	−	−
	Keller–Killiani test	−	−	−	−
Anthraquinone	Borntrager’s Test	−	−	−	−
	Modified Born. Test	−	−	−	−

(+) means present, (−) means absent.

**Table 4:** Total phenolic acids, total flavonoids, and total tannins of the different extracts of *C. calcitrapa*.

Extracts	Total Flavonoids (mg RTE/g)	Total Phenolic Acids (mg GAE/g)	Total Tannins (mg TAE/g)
Chloroform	305.27 ± 1.88	27.42 ± 0.29	12.02 ± 0.55
Ethyl acetate	414.43 ± 2.15	64.25 ± 0.96	26.01 ± 0.81
Methanol	425.93 ± 1.27	97.25 ± 0.73	27.52 ± 0.53
Aqueous	141.10 ± 1.31	17.25 ± 0.73	7.35 ± 0.56

Data are presented as Mean ± SE for 3 replicates (n = 3).

**Table 5:** Microbial sensitivity of different extractions against pathogenic tested microorganisms.

No.	Isolate Name	Diameter of Inhibition Zone (mm) by 100 µL/mL								
		AMC	Aqueous	Methanol	Ethyl Acetate	Chloroform	Aqueous Ext. 1	Chloroform Ext. 2	Ethyl Acetate Ext. 3	Methanol Ext. 4
1	*S. aureus*	10	ND	ND	ND	ND	27 ± 0.34 ^a^	31 ± 0.58 ^b^	32 ± 0.58 ^c^	33 ± 0.44 ^d^
2	*S. haemolyticus*	8	ND	ND	ND	ND	27 ± 0.35 ^a^	31 ± 0.56 ^b^	32 ± 0.59 ^c^	32 ± 0.46 ^c^
3	*E. faecalis*	ND	ND	ND	ND	ND	28 ± 0.22 ^a^	30 ± 0.25 ^b^	30 ± 0.21 ^b^	31 ± 0.31 ^c^
4	*E. coli*	ND	ND	ND	ND	ND	28 ± 0.21 ^a^	30 ± 0.27 ^b^	31 ± 0.34 ^c^	32 ± 0.43 ^d^
5	*K. pneumoniae*	11	ND	ND	ND	ND	28 ± 0.23 ^a^	31 ± 0.57 ^b^	31 ± 0.35 ^b^	32 ± 0.43 ^c^
6	*E. aerogeenes*	ND	ND	ND	ND	ND	28 ± 0.21 ^a^	29 ± 0.73 ^b^	30 ± 0.3 ^c^	31 ± 0.33 ^d^
7	*A. baumanii*	ND	ND	ND	ND	ND	28 ± 0.27 ^a^	29 ± 0.74 ^b^	30 ± 0.22 ^c^	31 ± 0.34 ^d^

ND, not detected, and data are presented as Mean ± SE for 3 replicates (n = 3). Indicating significantly different mean values (*p* < 0.05), distinct letters (a, b, c, and d) appear on bars at the same concertation.

**Table 6:** Minimum inhibitory concentrations and minimum lethal concentrations of different extracts of *C. calcitrapa* against different human pathogens.

**No.**	**MICs and MLCs of Aqueous Ext. by μg/mL**		**MICs and MLCs of Chloroform Ext. by μg/mL**	
	**MICs**	**MLCs**	**MICs**	**MLCs**
1	25 ± 0.43 ^b^	50 ± 0.52 ^a^	25 ± 0.42 ^a^	50 ± 0.5 ^a^
2	25 ± 0.44 ^b^	50 ± 0.51 ^a^	25 ± 0.4 ^a^	50 ± 0.52 ^a^
3	25 ± 0.41 ^b^	50 ± 0.53 ^a^	25 ± 0.42 ^a^	25 ± 0.72 ^b^
4	12.5 ± 0.75 ^c^	25 ± 0.73 ^b^	12.5 ± 0.74 ^b^	50 ± 0.51 ^a^
5	25 ± 0.42 ^b^	50 ± 0.55 ^a^	25 ± 0.41 ^a^	50 ± 0.52 ^a^
6	50 ± 0.53 ^a^	50 ± 0.53 ^a^	25 ± 0.43 ^a^	50 ± 0.51 ^a^
7	50 ± 0.55 ^a^	50 ± 0.52 ^a^	25 ± 0.41 ^a^	50 ± 0.53 ^a^
	**MICs and MLCs of Ethyl Acetate Ext. by μg/mL**		**MICs and MLCs of Methanol Ext. by** **μg/mL**	
	**MICs**	**MLCs**	**MICs**	**MLCs**
1	25 ± 0.43 ^a^	25 ± 0.4 ^a^	6.25 ± 0.9 ^b^	12.5 ± 0.7 ^b^
2	12.5 ± 0.72 ^b^	25 ± 0.43 ^a^	6.25 ± 0.92 ^b^	12.5 ± 0.69 ^b^
3	25 ± 0.41 ^a^	25 ± 0.41 ^a^	6.25 ± 0.93 ^b^	12.5 ± 0.7 ^b^
4	12.5 ± 0.7 ^b^	25 ± 0.4 ^a^	12.5 ± 0.72 ^a^	25 ± 0.44 ^a^
5	25 ± 0.4 ^a^	25 ± 0.4 ^a^	12.5 ± 0.7 ^a^	25 ± 0.41 ^a^
6	25 ± 0.42 ^a^	25 ± 0.43 ^a^	12.5 ± 0.7 ^a^	25 ± 0.42 ^a^
7	12.5 ± 0.71 ^b^	25 ± 0.41 ^a^	12.5 ± 0.72 ^a^	25 ± 0.43 ^a^

Where 1, *S. aureus*; 2, *S. haemolyticus*; 3, *E. faecalis*; 4, *E. coli*; 5, *K. pneumoniae*; 6, *E. aerogeenes*; and 7, *A. baumanii*. Data are presented as Mean ± SE for 3 replicates (n = 3). At the same concertation, various letters (a, b, and c) indicate that the mean values differ significantly (*p* ≤ 0.05).

## Data Availability

All authors declare that the data supporting the findings of this study are available within the article.
